# A New Pt(II) Complex with Anionic *s*-Triazine Based *NNO*-Donor Ligand: Synthesis, X-ray Structure, Hirshfeld Analysis and DFT Studies

**DOI:** 10.3390/molecules27051628

**Published:** 2022-03-01

**Authors:** Mezna Saleh Altowyan, Saied M. Soliman, Jamal Lasri, Naser E. Eltayeb, Matti Haukka, Assem Barakat, Ayman El-Faham

**Affiliations:** 1 Department of Chemistry, College of Science, Princess Nourah bint Abdulrahman University, P.O. Box 84428, Riyadh 11671, Saudi Arabia; msaltowyan@pnu.edu.sa; 2 Department of Chemistry, Faculty of Science, Alexandria University, P.O. Box 426, Ibrahimia, Alexandria 21321, Egypt; ayman.elfaham@alexu.edu.eg; 3 Department of Chemistry, Rabigh College of Science and Arts, King Abdulaziz University, Jeddah 21589, Saudi Arabia; nasertaha90@gmail.com; 4 Department of Chemistry, University of Jyväskylä, P.O. Box 35, FI-40014 Jyväskylä, Finland; matti.o.haukka@jyu.fi; 5 Department of Chemistry, College of Science, King Saud University, P.O. Box 2455, Riyadh 11451, Saudi Arabia

**Keywords:** Pt(II) complex, *s*-triazine, Hirshfeld, NBO, TD-DFT, X-ray

## Abstract

The reaction of PtCl_2_ with *s*-triazine-type ligand (**HTriaz**) (1:1) in acetone under heating afforded a new **[Pt(Triaz)Cl]** complex. Single-crystal X-ray diffraction analysis showed that the ligand (**HTriaz**) is an *NNO* tridentate chelate via two N-atoms from the *s*-triazine and hydrazone moieties and one oxygen from the deprotonated phenolic OH. The coordination environment of the Pt(II) is completed by one Cl^−1^ ion *trans* to the Pt-N_(hydrazone)_. Hirshfeld surface analysis showed that the most dominant interactions are the H···H, H···C and O···H intermolecular contacts. These interactions contributed by 60.9, 11.2 and 8.3% from the whole fingerprint area, respectively. Other minor contributions from the Cl···H, C···N, N···H and C···C contacts were also detected. Among these interactions, the most significant contacts are the O···H, H···C and H···H interactions. The amounts of the electron transfer from the ligand groups to Pt(II) metal center were predicted using NBO calculations. Additionally, the electronic spectra were assigned based on the TD-DFT calculations.

## 1. Introduction

*s-*triazine and their metal complexes have gained much attention for their properties and potential applications in many fields [1]. In the last decade, *s-*triazine and their complexes have been explored in the pharmaceutical field, catalytic process including Heck and Suzuki-Miyaura cross-coupling reactions, olefin polymerization, hydrogen transfer reactions, decarbonylation of ketones, asymmetric allylic alkylation, and some derivatives have been designed to develop photoelectronic materials [1]. Several ligands have been synthesized based on the *s-*triazine as a core structure and have been explored in coordination chemistry [1]. Mukherjee et al. constructed a complicated coordinated molecule by coordination-driven self-assembly of homometallic Pd/Pt-based *s*-triazine ligand as interlocked molecular cages [2]. Motloch et al. reported the synthesis of the Pt(II)/Pd(II) complex with *s*-triazine-type ligands for the purpose of hydrogen bonded/metal-coordination hybrid [3]. Another representative example was designed, synthesized and characterized by He et al. via self-assembly of supramolecular coordination complexes using platinum salt with two different types of pyridyl-derivatized ligands [4]. The photophysical properties of these supramolecular coordination complexes showed potential metal ion-responsive materials [4]. In the same field of photophysical study, a host–guest coordination cage has been assembled, and demonstrated a primary ultrafast excited dynamic process including excited-state energy and charge transfer. This tailored architecture was designed by the Han research group [5]. This fascinating *s*-triazine ligand has attracted great attention due to its several applications [6,7,8,9,10,11,12,13]. Mao et al. designed and synthesized two trigeminal star-like platinum complexes which stabilized hTel G4 with high selectivity and affinity, targeting telomerase inhibitors [14]. Additionally, some Pd(II)-*s*-triazine complexes have been constructed and assessed against breast cancer cell lines (MCF7 and MDA-MB-231) and have exhibited good potentials [15,16]. The design of new *s*-triazine-based ligands and their coordination modes with different metal centers is still a challenge [17,18,19]. Recently, Barakat et al. designed, synthesized and characterized a new hydrazono-*s*-triazine-based ligand and later explored the coordination chemistry of this ligand with a palladium(II) center. This study revealed that palladium coordinated via the *s*-triazine-type ligand as an *NNO*-donor [20]. Additionally, reaction of PdCl_2_ with 4,4′-(6-(3,5-dimethyl-1*H*-pyrazol-1-yl)-1,3,5-triazine-2,4-diyl)dimorpholine (**MPT**) and *N*-methyl-*N*-phenyl-4,6-di(1*H*-pyrazol-1-yl)-1,3,5-triazin-2-amine (**BPT**) ligands afforded the corresponding [Pd(MPT)Cl_2_] and [Pd(BPT)Cl]ClO_4_ tetracoordinated Pd(II) complexes. In these Pd(II) complexes, the *s*-triazine ligands worked as bidentate and tridentate chelates, respectively [16]. Both complexes were found to have improved anticancer activities against MDA-MB-231 and MCF-7 cell lines compared to the corresponding free ligands. On the other hand, the reaction of PdCl_2_ with 2,4-*bis*(3,5-dimethyl-1*H*-pyrazol-1-yl)-6-methoxy-1,3,5-triazine proceeded with partial hydrolysis of the ligand to 6-(3,5-dimethyl-1*H*-pyrazol-1-yl)-1,3,5-triazine-2,4(1*H*,3*H*)-dione (**HPT**) and the square planar complex [Pd(PT)Cl(H_2_O)]*H_2_O was obtained [15]. In addition, the Pd(II) complex was found to have almost equal activities against MDA-MB-231 and MCF-7 cell lines. Interestingly, the reaction of the same ligand with PtCl_2_ proceeded with complete hydrolysis of the ligand as indicated by the formation of [Pt(3,5-dimethyl-1*H*-pyrazole)_2_Cl_2_] [15].

**Figure 1:**
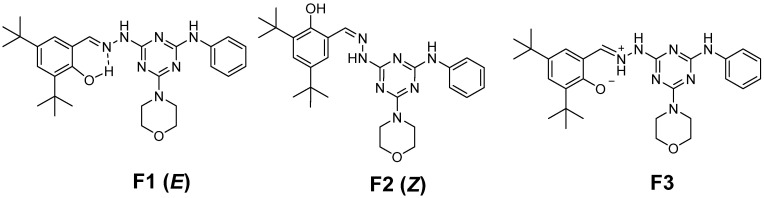
Structure of *s*-triazine hydrazone ligand (**HTriaz**).

During our study, we have explored the utility of the hydrazono-*s*-triazine-based ligand towards metalation with the divalent platinum ion to synthesize a new Pt(II) complex based on *s*-triazine hydrazone ligand (Figure 1). Its 3D molecular and supramolecular structures were elucidated by single-crystal X-ray diffraction and Hirshfeld analyses. The chemical insights of the Pt(II) complex have also been demonstrated.

## 2. Results and Discussion

### 2.1. [Pt(Triaz)Cl] Complex Synthesis and Chracterization

**Scheme 1:**
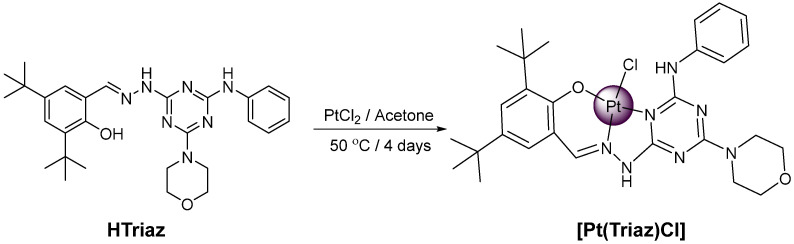
Synthesis of [Pt(Triaz)Cl] complex.

The Pt(II) complex **[Pt(Triaz)Cl]** was synthesized by reaction of (**HTriaz**) ligand with platinum (II) chloride (1:1) in acetone under heating (Scheme 1). The new Pt(II) complex was characterized by FT-IR, UV–Vis, single-crystal X-ray diffraction and CHNPt analyses. The reported structure by single-crystal X-ray diffraction agreed very well with the elemental analysis results. Additionally, the FT-IR spectra of **[Pt(Triaz)Cl]** exhibited vibrational characteristics of the functional groups, e.g., NH (3428 cm^−1^), aromatic C–H (3120 cm^−1^), aliphatic C–H (2957 and 2866 cm^−1^), C=N/C=C (1630 cm^−1^).

### 2.2. Crystal Structure Description

**Figure 2:**
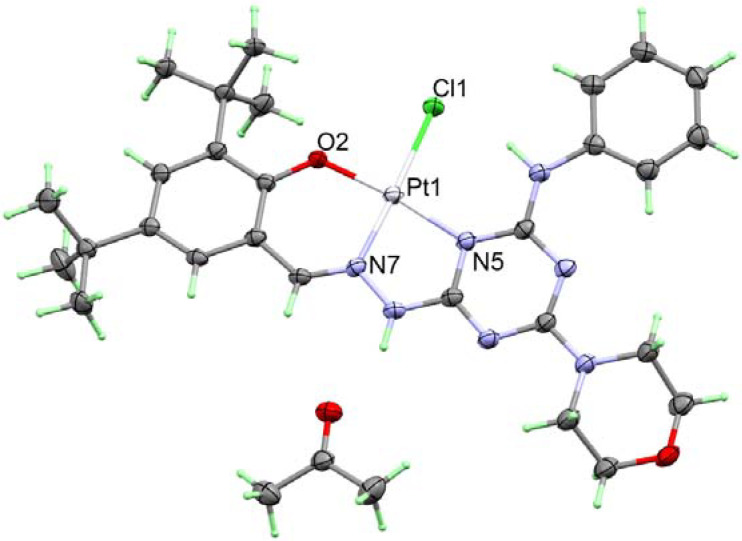

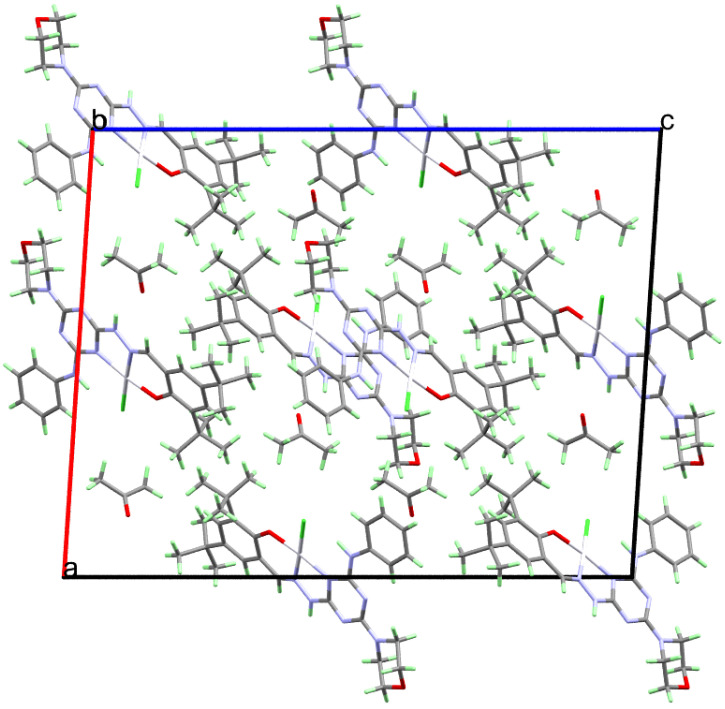
X-ray structure (**upper**) and packing view along *ac*-plane (**lower**) for **[Pt(Triaz)Cl]** complex.

The X-ray structure of **[Pt(Triaz)Cl]** including atom numbering and thermal ellipsoids drawn at 50% probability level is shown in Figure 2 (upper part). The **[Pt(Triaz)Cl]** complex crystallized in *I2/a* space group (Table S1; Supplementary data). The asymmetric unit comprised one **[Pt(Triaz)Cl]** complex unit and one acetone as a crystal solvent. The ligand (**Triaz^−1^**) is a *NNO* tridentate ligand. The donor atoms of this ligand are two nitrogen atoms from the *s*-triazine and the hydrazone fragments in addition to the phenolic oxygen atom. The coordination environment of the Pt(II) is completed by one Cl^−1^ *trans* to the Pt-N_(hydrazone)_. The Pt to donor atoms (N4, N7, O2 and Cl1) distances are 2.055(4), 1.945(4), 1.991(4) and 2.331(1) Å, respectively. The angle between the *trans*-bonds O2-Pt1-N4 and N7-Pt1-Cl1 are 173.35(16) and 177.01(13) Å, respectively (Table 1). The results are in good agreement with the X-ray structure of the structurally related **[Pd(Triaz)Cl]** complex [20].

On the other hand, the angles between the *cis*-bonds are in the range of 83.91(11)–102.58(13)°, indicating a distorted square planar coordination environment around the Pt(II). The structure of this complex showed one intramolecular N-H···O H-bond between the N–H group from the organic ligand as a H-bond donor and the carbonyl oxygen atom from the acetone molecule as H-bond acceptor. The hydrogen-acceptor and donor-acceptor distances are 2.028 and 2.777(7) Å, respectively, while the N6-H6···O3 angle is 141.6°. A view of packing along *ac*-plane is shown in the lower part of Figure 2.

### 2.3. Analysis of Molecular Packing

**Figure 3:**
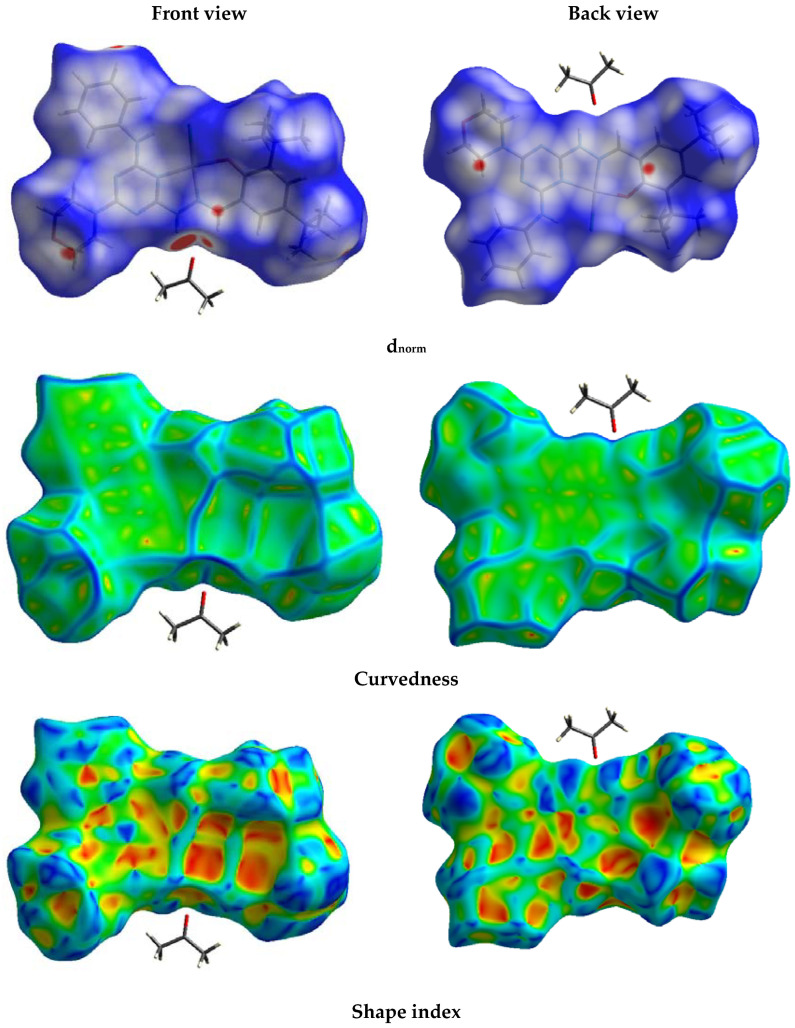
Hirshfeld surfaces of **[Pt(Triaz)Cl]**.

**Figure 4:**
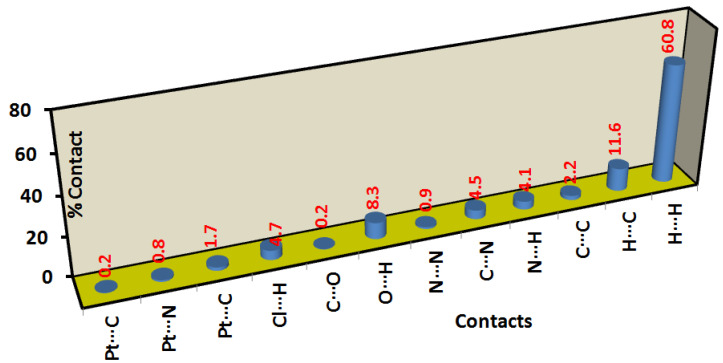
Percentages of intermolecular contacts in **[Pt(Triaz)Cl]**.

Hirshfeld surfaces mapped over d_norm_, shape index (SI) and curvedness for the studied complex are shown in Figure 3, while the different contacts and their contribution percentages in the molecular packing are present in Figure 4.

**Figure 5:**
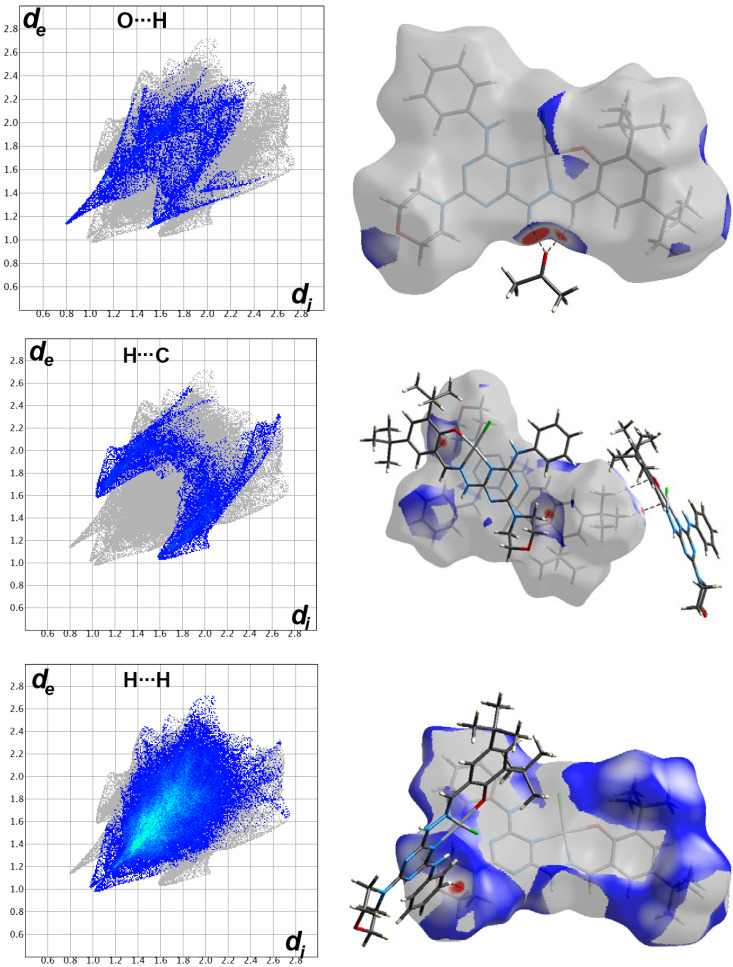
d_norm_ maps (**right**) and fingerprint plots (**left**) of the O···H, H···C and H···H contacts in **[Pt(Triaz)Cl]**.

As can be seen from Figure 4, the most dominant interactions are the H···H, H···C and O···H intermolecular contacts. These interactions contributed 60.9, 11.2, and 8.3% of the whole fingerprint area while the corresponding values for the **Pd(II)** complex are 60.6, 11.6, and 8.1, respectively. Other minor contributions from the Cl···H, C···N, N···H and C···C contacts were also detected. Generally, the most significant contacts are the O···H and H···C interactions. The latter belongs to the C-H···π interactions. In the corresponding **Pd(II)** complex, the O···H, H···H and H···C interactions are the most important. These intermolecular contacts appeared as red spots in d_norm_ and characterized by spikes in the fingerprint plots as shown in Figure 5. The O···H interactions appeared as one spike in the upper left part of the fingerprint plot due to the N–H···O (1.934 Å) and C–H···O (2.416 Å) interactions between the carbonyl group as hydrogen bond acceptor and the surface as hydrogen bond donor. On the other hand, the C–H···π interactions are characterized by two spikes with interaction distances ranges from 2.630 Å (H4A···C15) to 2.785 Å (H19B ···C16). In the corresponding **Pd(II)** complex, the O···H and H···C interactions are 1.839 and 2.608 Å, respectively which are slightly shorter than the corresponding values of the **[Pt(Triaz)Cl]** complex. In the former, all H···H interactions have long interaction distances while in the latter, most H···H interactions also have long interaction distances, except for the H11···H2B contact, which appeared as a red spot in the d_norm_. The H11···H2B contact distance is 2.003 Å. A summary of all contacts with shorter distances than the vdW radii sum of the interacting elements is listed in Table 2.

### 2.4. DFT Studies

**Figure 6:**
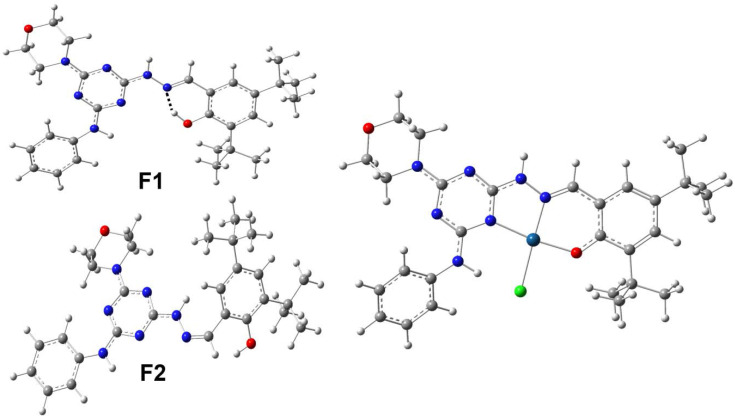
The optimized geometries of the two isomers (**F1** and **F2**) of the ligand **HTriaz** (**left**) and **[Pt(Triaz)Cl]** complex (**right**).

The optimized structures of **[Pt(Triaz)Cl]** and two possible geometrical isomers (**F1** (***E***) and **F2** (***Z***); Figure 1) of the free ligand are shown in Figure 6. The total energies of the ligand isomers are −1622.4327 and −1622.4126 a.u. for **F1** and **F2**, respectively. Hence, **F1** is the more stable than **F2** by 12.6019 kcal/mol. This result agreed with our previous studies [21]. The extra stability of **F1** could be attributed to the presence of intramolecular O–H···N hydrogen bond between the hydrazone nitrogen atom and the OH proton with hydrogen-acceptor and donor-acceptor distances of 1.729 and 2.608 Å, respectively. Another possible isomer in which the labile proton is bonded to the Schiff base nitrogen atom leading to a zwitterion species is abbreviated in Figure 1 as **F3**. The structure of **F3** was optimized using the same level of theory. Interestingly, the geometry optimization ended to the same optimized structure of **F1** indicating that the form **F1** is more favored than the NH zwitter ionic form **F3**. Additionally, the proton affinity of **Triaz¯** was calculated based on the enthalpy change (*ΔH*) of the reaction **Triaz^−^+H^+^→HTriaz** to be 353.06 kcal/mol. On the other hand, the Pt(II) affinity **Triaz^−^** was calculated to be 589.111 kcal/mol. In this regard, one could conclude that the higher affinity of **Triaz^−^** to the Pt(II) could be attributed to the chelate effect where the coordination between the Pt(II) ion and the tridentate **Triaz^−^** ligand lead to the formation of two chelate rings which could be the driving force for the deprotonation of the **HTriaz** and breaking the intramolecular O–H···N hydrogen bonding interaction of **F1**.

**Figure 7:**
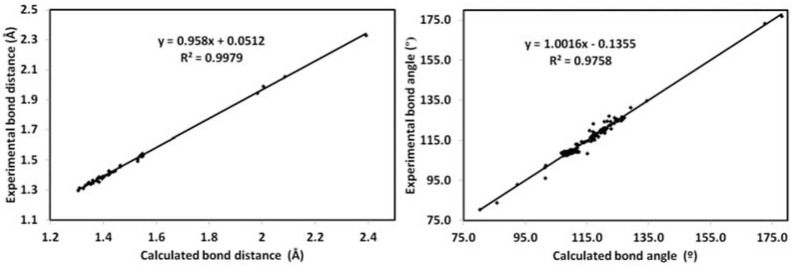
Correlations between the calculated and experimental bond distances (**left**) and angles (**right**).

On the other hand, the optimized structure of the **[Pt(Triaz)Cl]** complex agreed very well with the experimental X-ray structure (Table S2, Supplementary data). In addition, good correlations were obtained between the calculated and experimental geometric parameters. The correlation coefficients for bond distances and angles are 0.9979 and 0.9758, respectively (Figure 7). The ligand and its Pt(II) complex are polar compounds where the calculated dipole moments are 7.933 and 2.289 Debye, respectively. It is clear that complexation of the ligand with Pt(II) decreased the polarity of the system.

The interaction between Pt(II) as a Lewis acid and ligand as a Lewis base affect the net charge at both fragments. The calculated charges at Pt, Cl, and the anionic ligand are depicted in Table 3. The charge at the Pt(II) is changed to +0.5 instead of +2.0 due to the large electron density transferred from the ligand groups. The amount of negative electron density transferred from the ligand groups are 0.56 and 0.95 e for the **Cl^−1^** and **Triaz^−1^**, respectively.

### 2.5. UV–Vis Spectra

**Figure 8:**
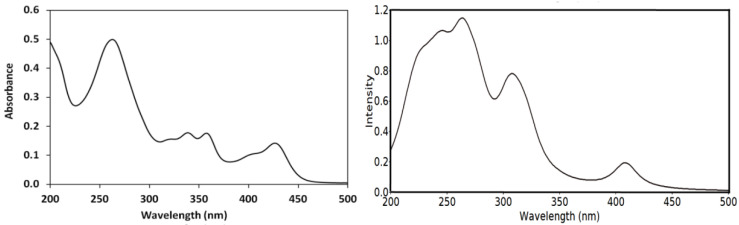
The experimental (**left**) and calculated (**right**) UV–Vis spectra of the studied **[Pt(Triaz)Cl]** complex.

**Figure 9:**
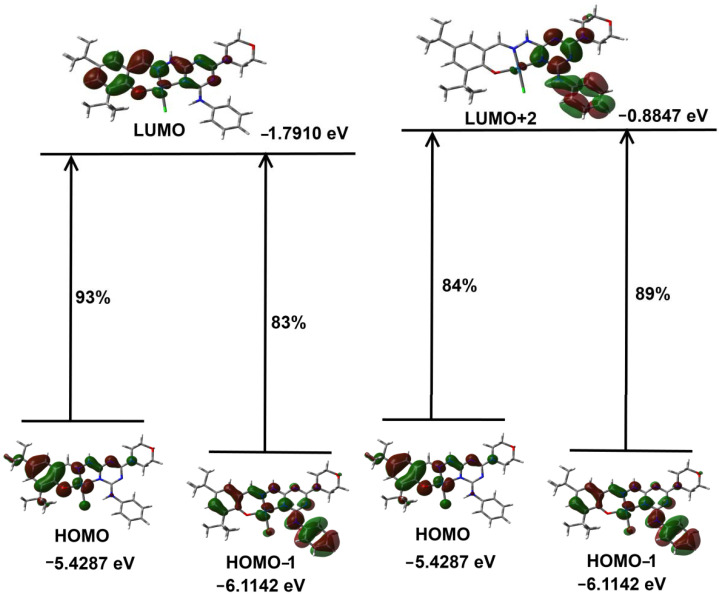
The MOs included in the electronic transitions of the studied **[Pt(Triaz)Cl]** complex.

The experimental and calculated UV–Vis spectra of the studied Pt(II) complex in ethanol as solvent are presented in Figure 8. The longest wavelength band was observed experimentally at 427 nm. The TD-DFT calculations predicted this band at 409 nm with oscillator strength of 0.1646. This electronic transition was assigned to HOMO→LUMO (93%) excitation. In addition, the TD-DFT calculations predicted intense absorptions at 322 nm (exp. 338 nm) and 305 nm (exp. 320 nm) with oscillator strengths of 0.2102 and 0.2196, respectively. These electronic transition bands were assigned to H−1→LUMO (83%) and HOMO→L+2 (84%), respectively. Experimentally, the region below 300 nm showed an intense absorption at 261 nm, which is calculated at 266 nm (f = 0.3628). This band was assigned to H−1→L+2 (89%) excitation. Presentation of molecular orbitals (MOs) included in these electronic transitions are shown in Figure 9. Theoretically, an absorption band and a shoulder were predicted at 247 nm (f = 0.1883) and 226 nm (f = 0.1040), respectively. The former was assigned to the mixed H−3→L+2 (56%) and HOMO→L+5 (11%) transitions while the latter was assigned for H−3→L+3(26%) and HOMO→L+6 (17%)/L+7 (35%) transitions.

## 3. Materials and Methods

### 3.1. Materials and Methods

Chemicals were purchased from Sigma–Aldrich (Chemie GmbH, 82024 Taufkirchen, Germany). The CHN analyses were determined using Perkin–Elmer 2400 instrument (PerkinElmer, Inc.940 Winter Street, Waltham, MA, USA). Pt content was determined using a Shimadzu atomic absorption spectrophotometer (AA-7000 series, Shimadzu, Ltd., Japan). FT-IR spectrum was assessed on a Perkin–Elmer 1000 FT-IR spectrometer, Waltham, MA, USA (Figure S1). The UV–Vis electronic spectrum of the Pt(II) complex at 3.0 × 10^−4^ mol L^−1^ in absolute ethanol as solvent was carried out using a UV–Vis spectrophotometer (Perkin–Elmer Lambda 35, Waltham, MA, USA) in 1 cm cell in the spectral range of 200–500 nm. Mass spectrum was recorded on JMS-600 H JEOL spectrometer (JEOL Ltd., Tokyo, Japan). ^1^H and ^13^C NMR spectra of **[Pt(Triaz)Cl]** were recorded on DMSO-*d*_6_ using a JEOL 500 MHz spectrometer (JEOL Ltd., Tokyo, Japan) at room temperature.

### 3.2. Synthesis of the Ligand (HTriaz)

The ligand (**HTriaz**) has been prepared using our published method [20,22] and the NMR spectral data agreed with the reported data [20].

### 3.3. Synthesis of [Pt(Triaz)Cl] Complex

The (**HTriaz**) ligand (60.0 mg, 0.119 mmol) was dissolved in 30 mL of acetone then PtCl_2_ (31.6 mg, 0.119 mmol) was added. The reaction mixture was heated at 50 °C for 4 days. Then, the resulting solution mixture was filtered, and the filtrate was left for slow evaporation at room temperature to afford the final product **[Pt(Triaz)Cl]** as reddish-brown block crystals. Yield; C_31_H_42_ClN_7_O_3_Pt 79%; Anal. Calcd. for: C, 47.06; H, 5.35; N, 12.39; Pt, 24.65. Found: C, 47.24; H, 5.29; N, 12.20; Pt, 24.46. FT-IR (KBr) cm^−1^: 3428 (NH), 3263, 3120, 2957, 2866 (C-H), 1540 and 1630 (C=N and C=C) (Figure S1; Supplementary data); ^1^H NMR (500 MHz, DMSO-*d*_6_, ppm): *δ* 1.24 (s, 9H, 3CH_3_), 1.37 (s, 9H, 3CH_3_), 3.64 (t, 4H, *J* = 4.0 Hz, 2CH_2_ (morpholine ring), 3.71 (t, 4H, *J* = 3.6 Hz, 2CH_2_ (morpholine ring), 7.14 (t, 1H, *J* = 6.8 Hz, C_6_H_5_), 7.30 (d, 1H, *J* = 2.0 Hz, C_6_H_5_), 7.30–7.35 (m, 3H, C_6_H_5_ and C_6_H_2_), 7.63-7.57 (m, 3H, C_6_H_5_ and C_6_H_2_ and CH=N), 8.46 (s, 1H, NH), 10.91 (s, 1H, NH) (Figure S2; Supplementary data). ^13^C NMR (126 MHz, DMSO-*d*_6_) δ 194.92, 178.15, 166.74, 164.72, 143.14, 141.50, 140.00, 138.81, 133.36, 131.26, 130.51, 128.42, 125.73, 125.09, 124.80, 123.88, 123.08, 122.93, 116.34, 116.17, 111.63, 74.32, 74.26, 62.38, 54.28, 49.14, 47.13, 36.54 (Figure S3; Supplementary data).

### 3.4. X-ray Structure Determinations

The details of the crystal structure determination are found in Table S1 and all technical experiments are provided in the supplementary materials [23,24,25,26,27].

### 3.5. Hirshfeld and DFT Calculations

Crystal Explorer 17.5 [28] was used to perform the analysis of molecular packing. Details of DFT and TD-DFT calculations [29,30,31,32,33,34] as well as proton affinity [35] are given in supplementary data.

## 4. Conclusions

A novel Pt(II) complex **[Pt(Triaz)Cl]** with tridentate *NNO*-donor ligand-based *s*-triazine scaffold was achieved. The chemical structure of **[Pt(Triaz)Cl]** was confirmed by CHNPt analyses and single-crystal X-ray diffraction. The Pt(II) coordination environment is distorted square planar. The structure of this complex showed one intramolecular N-H···O hydrogen bond between the N–H group from the organic ligand as a hydrogen bond donor and the carbonyl oxygen atom from the acetone molecule as a hydrogen bond acceptor. The supramolecular structure of the studied Pt(II) complex is analyzed using Hirshfeld calculations. Additionally, the calculated UV–Vis spectra were assigned based on the results of the TD-DFT calculations. The natural charges were calculated, and the results indicated that the amount of the electron transfer from the **Cl^−1^** and **Triaz^−1^** is 0.56 and 0.95 e, respectively.

## Figures and Tables

**Table 1:** **[Pt(Triaz)Cl]** complex bond lengths [Å] and angles [°].

**Atoms**	**Distance**	**Atoms**	**Distance**
Pt1-N7	1.945(4)	Pt1-N4	2.055(4)
Pt1-O2	1.991(4)	Pt1-Cl1	2.3308(13)
**Atoms**	**Angle**	**Atoms**	**Angle**
N7-Pt1-O2	93.09(16)	N7-Pt1-Cl1	177.01(13)
N7-Pt1-N4	80.42(18)	O2-Pt1-Cl1	83.91(11)
O2-Pt1-N4	173.35(16)	N4-Pt1-Cl1	102.58(13)

**Table 2:** Short interactions and their contact distances in **[Pt(Triaz)Cl]**.

Contact	Distance	Contact	Distance
O3···H1	2.416	H4A···C15	2.630
O3···H6	1.934	H4A ···C28	2.777
H19B ···C16	2.785	H11···C2	2.689
H20B ···C14	2.656	H11···H2B	2.003

**Table 3:** The calculated charge at Pt, Cl and the anionic ligand.

Atom/Group	Optimized	X-ray
**Pt**	0.4998	0.4857
**Cl**	−0.4410	−0.4402
**Triaz**	−0.0588	−0.0455

## Data Availability

Not applicable.

## Supplementary Materials

The following supporting information can be downloaded online. Table S1: Crystal data and structure refinement for [Pt(Triaz)Cl]; Table S2. The calculated geometric parameters of [Pt(Triaz)Cl]; Figure S1: FT-IR spectra of the studied Pt(II) complex; Figure S2: ^1^H NMR spectra of the studied Pt(II) complex; Figure S3: ^13^C NMR spectra of the studied Pt(II) complex.

## Sample Availability

Samples of the compound **[Pt(Triaz)Cl]** is available from the authors.

## References

[1] Barakat A., El-Faham A., Haukka M., Al-Majid A.M., Soliman S.M. (2021). *s*-Triazine pincer ligands: Synthesis of their metal complexes, coordination behavior, and applications. Appl. Organomet. Chem..

[2] Kumar A., Mukherjee P.S. (2020). Multicomponent Self-Assembly of PdII/PtII Interlocked Molecular Cages: Cage-to-Cage Conversion and Self-Sorting in Aqueous Medium. Chem. Eur. J..

[3] Motloch P., Hunter C.A. (2021). Stimuli-Responsive Self-Sorting Hybrid Hydrogen-Bonded/Metal-Coordinated Cage. Chem. Eur. J..

[4] He Z., Li M., Que W., Stang P.J. (2017). Self-assembly of metal-ion-responsive supramolecular coordination complexes and their photophysical properties. Dalton Trans..

[5] Zhang R.L., Yang Y., Yang S.Q., Han K.L. (2018). Unveiling excited state energy transfer and charge transfer in a host/guest coordination cage. Phys. Chem. Chem. Phys..

[6] Fu H.L.K., Leung S.Y.L., Yam V.W.W. (2017). A rational molecular design of triazine-containing alkynylplatinum (II) terpyridine complexes and the formation of helical ribbons via Pt⋯ Pt, π–π stacking and hydrophobic–hydrophobic interactions. Chem. Commun..

[7] Liu N., Lin T., Wu M., Luo H.K., Huang S.L., Hor T.A. (2019). Suite of Organoplatinum (II) triangular metallaprism: Aggregation-induced emission and coordination sequence induced emission tuning. J. Am. Chem. Soc..

[8] Marzo T., Cirri D., Ciofi L., Gabbiani C., Feis A., Di Pasquale N., Stefanini M., Biver T., Messori L. (2018). Synthesis, characterization and DNA interactions of [Pt_3_(TPymT)Cl_3_], the trinuclear platinum (II) complex of the TPymT ligand. J. Inorg. Biochem..

[9] Ismail A.M., El Sayed S.A., Butler I.S., Mostafa S.I. (2020). New Palladium (II), Platinum (II) and Silver (I) complexes of 2-amino-4, 6-dithio-1,3,5-triazine; synthesis, characterization and DNA binding properties. J. Mol. Struct..

[10] Yetim N.K., Sarı N. (2019). Novel dendrimers containing redox mediator: Enzyme immobilization and applications. J. Mol. Struct..

[11] Paul L.E., Therrien B., Furrer J. (2018). The complex-in-a-complex cation [Pt (acac) 2-(p-cym)_6_Ru_6_(tpt)_2_(dhnq)_3_] ^6+^: Its stability towards biological ligands. Inorg. Chim. Acta.

[12] Asman P.W. (2018). Kinetics and mechanistic study of polynuclear platinum (II) polypyridyl complexes; A paradigm shift in search of new anticancer agents. Inorg. Chim. Acta.

[13] Zhang W., Wang J., Xu Y., Li W., Shen W. (2017). Fine tuning phosphorescent properties of platinum complexes via different N-heterocyclic-based C-N-N ligands. J. Organomet. Chem..

[14] Zheng X.H., Mu G., Zhong Y.F., Zhang T.P., Cao Q., Ji L.N., Zhao Y., Mao Z.W. (2016). Trigeminal star-like platinum complexes induce cancer cell senescence through quadruplex-mediated telomere dysfunction. Chem. Comm..

[15] Lasri J., Haukka M., Al-Rasheed H.H., Abutaha N., El-Faham A., Soliman S.M. (2021). Synthesis, structure and in vitro anticancer activity of Pd (II) complex of pyrazolyl-*s*-triazine ligand; A new example of metal-mediated hydrolysis of *s*-triazine pincer ligand. Crystals.

[16] Lasri J., Al-Rasheed H.H., El-Faham A., Haukka M., Abutaha N., Soliman S.M. (2020). Synthesis, structure and in vitro anticancer activity of Pd (II) complexes of mono-and bis-pyrazolyl-*s*-triazine ligands. Polyhedron.

[17] Soliman S.M., Albering J.H., Sholkamy E.N., El-Faham A. (2020). Mono-and penta-nuclear self-assembled silver (I) complexes of pyrazolyl *s*-triazine ligand; synthesis, structure and antimicrobial studies. Appl. Organomet. Chem..

[18] Soliman S.M., El-Faham A. (2019). Synthesis and structure diversity of high coordination number Cd (II) complexes of large *s*-triazine bis-Schiff base pincer chelate. Inorg. Chim. Acta.

[19] Soliman S.M., El-Faham A., Elsilk S.E., Farooq M. (2018). Two heptacoordinated manganese (II) complexes of giant pentadentate *s*-triazine bis-Schiff base ligand: Synthesis, crystal structure, biological and DFT studies. Inorg. Chim. Acta.

[20] Soliman S.M., Lasri J., Haukka M., Elmarghany A., Al-Majid A.M., El-Faham A., Barakat A. (2020). Synthesis, X-ray structure, Hirshfeld analysis, and DFT studies of a new Pd (II) complex with an anionic *s*-triazine NNO donor ligand. J. Mol. Struct..

[21] Barakat A., El-Senduny F.F., Almarhoon Z., Al-Rasheed H.H., Badria F.A., Al-Majid A.M., Ghabbour H.A., El-Faham A. (2019). Synthesis, X-ray crystal structures, and preliminary antiproliferative activities of new s-triazine-hydroxybenzylidene hydrazone derivatives. J. Chem..

[22] Al-Rasheed H.H., Al Alshaikh M., Khaled J.M., Alharbi N.S., El-Faham A. (2016). Ultrasonic irradiation: Synthesis, characterization, and preliminary antimicrobial activity of novel series of 4,6-disubstituted-1,3,5-triazine containing hydrazone derivatives. J. Chem..

[23] Otwinowski Z., Minor W., Carter C.W., Sweet J. (1997). Processing of X-ray Diffraction Data Collected in Oscillation Mode. Methods in Enzymology, Volume 276, Macromolecular Crystallography, Part A.

[24] Sheldrick G.M. (2012). SADABS—Bruker Nonius Scaling and Absorption Correction.

[25] Sheldrick G.M. (2015). Crystal Structure Refinement with SHELXL. Acta Cryst. C.

[26] Hübschle C.B., Sheldrick G.M., Dittrich B. (2011). *ShelXle*: A Qt graphical user interface for *SHELXL*. J. Appl. Cryst..

[27] Rikagu Oxford Diffraction (2020). CrysAlisPro.

[28] Turner M.J., McKinnon J.J., Wolff S.K., Grimwood D.J., Spackman P.R., Jayatilaka D., Spackman M.A. Crystal Explorer17 (2017) University of Western Australia. https://crystalexplorer.scb.uwa.edu.au/.

[29] Frisch M.J., Trucks G.W., Schlegel H.B., Scuseria G.E., Robb M.A., Cheeseman J.R., Scalmani G., Barone V., Mennucci B., Petersson G.A. (2009). GAUSSIAN 09.

[30] Dennington R., Keith T., Millam J. (2007). GaussView.

[31] Reed A.E., Curtiss L.A., Weinhold F. (1988). Intermolecular interactions from a natural bond orbital, donor-acceptor viewpoint. Chem. Rev..

[32] Marten B., Kim K., Cortis C., Friesner R.A., Murphy R.B., Ringnalda M.N., Sitkoff D., Honig B. (1996). New model for calculation of solvation free energies:  Correction of self-consistent reaction field continuum dielectric theory for short-range hydrogen-bonding effects. J. Phys. Chem..

[33] Tannor D.J., Marten B., Murphy R., Friesner R.A., Sitkoff D., Nicholls A., Ringnalda M., Goddard W.A., Honig B. (1994). Accurate first principles calculation of molecular charge distributions and solvation energies from ab initio quantum mechanics and continuum dielectric theory. J. Am. Chem. Soc..

[34] Scalmani G., Frisch M.J., Mennucci B., Tomasi J., Cammi R., Barone V. (2006). Geometries and properties of excited states in the gas phase and in solution: Theory and application of a time-dependent density functional theory polarizable continuum model. J. Chem. Phys..

[35] Moser A., Range K., York D.M. (2010). Accurate proton affinity and gas-phase basicity values for molecules important in biocatalysis. J. Phys. Chem. B.

