# Supplementary material for: A New Pt(II) Complex with Anionic s-Triazine Based NNO-Donor Ligand: Synthesis, X-ray Structure, Hirshfeld Analysis and DFT Studies
Source: Molecules. 2022 Mar 1;27(5):1628. doi: 10.3390/molecules27051628 (PMC8911880; doi:10.3390/molecules27051628)
Supplement: Supplementary file 1 [file molecules-27-01628-s001.zip › molecules-1566817-supplementary.pdf]

molecules

MDPI

Article

A New Pt(II) Complex with Anionic *s*-Triazine Based *NNO*-Donor Ligand: Synthesis, X-Ray Structure, Hirshfeld Analysis and DFT Studies

Mezna Saleh Altowyan[1], Saied M. Soliman[2,*], Jamal Lasri[3,*], Naser E. Eltayeb[3], Matti Haukka[4], Assem Barakat[5,*] and Ayman El Faham[2]

[1] Department of Chemistry, College of Science, Princess Nourah bint Abdulrahman University, P.O. Box 84428, Riyadh 11671, Saudi Arabia; msaltowyan@pnu.edu.sa (M.S.A.)
[2] Department of Chemistry, Faculty of Science, Alexandria University, P.O. Box 426, Ibrahimia, Alexandria 21321, Egypt; ayman.elfaham@alexu.edu.eg (A.E.F.)
[3] Department of Chemistry, Rabigh College of Science and Arts, King Abdulaziz University, Jeddah 21589, Saudi Arabia; nasertaha90@gmail.com (N.E.E.).
[4] Department of Chemistry, University of Jyväskylä, P.O. Box 35, FI-40014 Jyväskylä, Finland; matti.o.haukka@jyu.fi
[5] Department of Chemistry, College of Science, King Saud University, P.O. Box 2455, Riyadh 11451, Saudi Arabia.
* Correspondence: saeed.soliman@alexu.edu.eg (S.M.S.), jlasri@kau.edu.sa (J.L.), ambarakat@ksu.edu.sa (A.B.)

Supplementary information

X-Ray structure determinations

The crystal of **[Pt(Triaz)Cl]** was immersed in cryo-oil, mounted in a loop, and measured at a temperature of 120 K. The X-ray diffraction data was collected on a Rigaku Oxford Diffraction Supernova diffractometer using Cu Kα radiation. The *CrysAlisPro* [S1] software package was used for cell refinement and data reduction. A Multi-scan, absorption correction (*CrysAlisPro* [S1]) was applied to the intensities before structure solution. Structure was solved by intrinsic phasing (*SHELXT* [S2]) method. Structural refinement was carried out using *SHELXL* [S3] software with *SHELXLE* [S4] graphical user interface. The NH hydrogen atoms were located from the difference Fourier map but constrained to ride on their parent nitrogen with U_{iso} = 1.5 U_{eq}(parent atom). All other hydrogen atoms were positioned geometrically and constrained to ride on their parent atoms, with C-H = 0.95 -1.00 Å and U_{iso} = 1.2-1.5·U_{eq}(parent atom).

Hirshfeld surface analysis

The topology analyses were performed using Crystal Explorer 17.5 program [S5] in order to analyze the different intermolecular interactions in the crystal structure of the studied Pt(II) complex.

Computational methods

All DFT calculations were performed using Gaussian 09 software package [S6] utilizing B3LYP method. The 6-31G(d,p) and LANL2DZ basis sets were used for nonmetal atoms and Pt, respectively. Natural bond orbital analyses were performed using NBO 3.1 program as implemented in the Gaussian 09W package [S7]. The self-consistent reaction filed (SCRF) method [S8-S9] was used to model the solvent effects when calculated the optimized geometry in solution. Then the electronic spectra of the fifty excited states for the studied Pt(II) complex were calculated using TD-DFT calculations [S10]. In addition, the structure of the **Triaz$^-$ and [Pt(Triaz)]$^+$** were optimized in order to compute the proton and Pt(II) affinities of the **Triaz$^-$** ligand anion, respectively [S11]. The proton and

Pt(II) affinities of **Triaz⁻** are calculated using the negative of the enthalpy change (*ΔH*) of the reactions **$Triaz^{-}$+H^{+}→HTriaz** and **$Triaz^{-}$+Pt^{2+}→[PtTriaz]$^{+}$**, respectively [S11].

References

[S1] Rikagu Oxford Diffraction, *CrysAlisPro*, Agilent Technologies inc., 2020, Yarnton, Oxfordshire, England.
[S2] Sheldrick, G. M. *SADABS - Bruker Nonius scaling and absorption correction -*, Bruker AXS, Inc.: Madison, Wisconsin, USA, 2012.
[S3] Sheldrick, G. M. Crystal Structure Refinement with SHELXL. *Acta Cryst.C.* **2015**, *C71*, 3–8.

[S4] Hübschle, C. B.; Sheldrick, G. M.; Dittrich, B. *ShelXle*: a Qt graphical user interface for *SHELXL*. *J. Appl. Cryst.* **2011**, *44*, 1281–1284.
[S5] Turner, M. J., McKinnon, J. J., Wolff, S. K., Grimwood, D. J., Spackman, P. R., Jayatilaka, D., & Spackman, M. A. Crystal Explorer17 (2017) University of Western Australia. https://crystalexplorer.scb.uwa.edu.au/
[S6] Frisch, M.J.; Trucks, G.W.; Schlegel, H.B.; Scuseria, G.E.; Robb, M.A.; Cheeseman, J.R.; Scalmani, G.; Barone, V.; Mennucci, B.; Petersson, G.A.; *et al. GAUSSIAN 09*; Revision A02; Gaussian Inc.: Wallingford, CT, USA, 2009; *GaussView*; Version 4.1; Dennington II, R., Keith, T., Millam, J., Eds.; Semichem Inc.: Shawnee Mission, KS, USA, 2007.
[S7] Reed, A.E.; Curtiss, L.A.; Weinhold, F. Intermolecular interactions from a natural bond orbital, donor-acceptor viewpoint. *Chem. Rev.* **1988**, *88*, 899–926.
[S8] Marten, B.; Kim K.; Cortis, C.; Friesner, R. A.; Murphy, R. B.; Ringnalda, M. N.; Sitkoff, D.; Honig, B. New Model for Calculation of Solvation Free Energies: Correction of Self-Consistent Reaction Field Continuum Dielectric Theory for Short-Range Hydrogen-Bonding Effects, *J. Phys. Chem.* **1996,** *100,* 11775–11765.
[S9] Tannor, D.J.; Marten, B.; Murphy, R.; Friesner, R.A.; Sitkoff, D.; Nicholls, A.; Ringnalda, M.; Goddard, W.A.; Honig, B. Accurate first principles calculation of molecular charge distributions and solvation energies from ab initio quantum mechanics and continuum dielectric theory. *J. Am. Chem. Soc.* **1994**, *116*, 11875–11882.
[S10] Scalmani, G.; Frisch, M. J.; Mennucci, B.; Tomasi, J.; Cammi, R.; Barone, V. Geometries and properties of excited states in the gas phase and in solution: Theory and application of a time-dependent density functional theory polarizable continuum model. *J. Chem. Phys.*, **2006**, *124*, 1–15.
[S11] A. Moser, K. Range, D. M. York, Accurate proton affinity and gas-phase basicity values for molecules important in biocatalysis. *J. Phys. Chem. B.* **2010**, *114,* 13911–13921.

Table S1. Crystal data and structure refinement for **[Pt(Triaz)Cl]**.

	**[Pt(Triaz)Cl]**.
CCDC	2132273
empirical formula	$C_{31}H_{42}ClN_7O_3Pt$
fw	791.25
temp (K)	120(2) K
λ(Å)	1.54184 Å
cryst syst	Monoclinic
space group	I2/a
a (Å)	22.9177(4)
b (Å)	9.9678(2)
c (Å)	29.0216(9)
β (deg)	93.698
V (Å^3)	6615.9(3) Å^3
Z	8
ρ_{calc} (Mg/m^3)	1.589 Mg/m^3
μ(Mo Kα) (mm^{-1})	9.018 mm^{-1}
No. reflns.	25659
Unique reflns.	6899
Completeness to θ=67.684°	100.0%
GOOF (F^2)	1.088
R_{int}	0.0446
R_1[a] ($I \geq 2\sigma$)	0.0394
wR_2[b] ($I \geq 2\sigma$)	0.0990

[a] $R1 = \Sigma||F_o| - |F_c||/\Sigma|F_o|$. [b] $wR_2 = \{\Sigma[w(F_o^2 - F_c^2)^2]/ \Sigma[w(F_o^2)^2]\}^{1/2}$

Table S2. The calculated geometric parameters of **[Pt(Triaz)Cl]**[a].

Parameter	Calc	Exp	Parameter	Calc	Exp
R(1-2)	2.391	2.331	A(2-1-4)	85.7	83.9
R(1-4)	2.004	1.991	A(2-1-8)	101.6	102.6
R(1-8)	2.086	2.056	A(2-1-12)	178.1	177.0
R(1-12)	1.981	1.944	A(4-1-8)	172.7	173.4
R(3-16)	1.42	1.43	A(4-1-12)	92.4	93.1
R(3-19)	1.422	1.406	A(1-4-75)	126.3	125.3
R(4-75)	1.31	1.315	A(1-4-70)	122.1	127.0
R(5-26)	1.345	1.353	A(1-4-72)	121.5	124.6
R(5-27)	1.333	1.332	A(8-1-12)	80.3	80.4
R(7-27)	1.342	1.347	A(1-8-27)	134.4	134.7
R(7-28)	1.417	1.403	A(1-8-39)	111.6	111.1
R(8-27)	1.384	1.355	A(1-12-10)	113.1	114.6
R(8-39)	1.359	1.367	A(1-12-40)	126.2	126.7
R(9-26)	1.352	1.342	A(16-3-19)	111.1	110.2
R(9-39)	1.325	1.314	A(3-16-13)	111.3	113.2
R(10-12)	1.377	1.377	A(3-16-17)	106.7	108.9
R(10-39)	1.361	1.35	A(3-16-18)	110.3	108.9
R(12-40)	1.305	1.298	A(3-19-20)	110.2	108.8
R(13-16)	1.53	1.493	A(3-19-21)	106.7	108.9
R(13-25)	1.463	1.465	A(3-19-22)	111.5	113.3
R(19-22)	1.53	1.507	A(4-75-42)	124.7	124.5
R(22-25)	1.464	1.456	A(4-75-61)	117.2	117.2
R(25-26)	1.356	1.346	A(75-4-70)	101.3	101.5
R(28-29)	1.402	1.388	A(75-4-72)	101.5	96.2
R(28-37)	1.404	1.395	A(26-5-27)	117.5	115.3
R(29-31)	1.395	1.383	A(5-26-9)	124.7	125.5
R(31-33)	1.395	1.377	A(5-26-25)	117.7	117.0
R(33-35)	1.396	1.391	A(5-27-7)	120.7	119.8
R(35-37)	1.393	1.383	A(5-27-8)	122.6	124.3
R(40-42)	1.426	1.424	A(6-7-27)	115.7	119.8
R(42-43)	1.425	1.416	A(6-7-28)	115.1	108.6
R(42-75)	1.436	1.424	A(27-7-28)	129.1	131.4
R(43-45)	1.374	1.365	A(7-27-8)	116.7	115.9
R(45-46)	1.538	1.527	A(7-28-29)	123.9	126.3
R(45-59)	1.421	1.405	A(7-28-37)	116.4	114.9
R(46-47)	1.54	1.535	A(27-8-39)	114.0	114.1
R(46-51)	1.547	1.546	A(8-39-9)	127.1	126.2
R(46-55)	1.547	1.526	A(8-39-10)	116.1	116.7
R(59-61)	1.384	1.39	A(26-9-39)	114.0	114.3
R(61-62)	1.545	1.54	A(9-26-25)	117.6	117.5
R(61-75)	1.444	1.428	A(9-39-10)	116.7	117.1
R(62-63)	1.543	1.528	A(11-10-12)	120.6	124.4
R(62-67)	1.549	1.53	A(11-10-39)	119.4	118.7
R(62-71)	1.549	1.533	A(12-10-39)	118.7	116.8

	A(10-12-40)	120.7	118.6
	A(12-40-41)	116.8	117.7
	A(12-40-42)	126.0	124.9
	A(14-13-15)	108.7	108.2
	A(14-13-16)	111.3	109.7
	A(14-13-25)	108.9	109.6
	A(15-13-16)	109.2	109.7
	A(15-13-25)	109.0	109.7
	A(16-13-25)	109.5	109.9
	A(13-16-17)	110.2	108.9
	A(13-16-18)	109.6	108.9
	A(13-25-22)	114.5	114.6
	A(13-25-26)	122.8	120.8
	A(17-16-18)	108.6	107.7
	A(20-19-21)	108.6	107.8
	A(20-19-22)	109.5	109.0
	A(21-19-22)	110.2	108.9
	A(19-22-23)	109.4	110.1
	A(19-22-24)	111.1	110.0
	A(19-22-25)	109.4	108.6
	A(23-22-24)	108.8	108.4
	A(23-22-25)	109.2	109.9
	A(24-22-25)	108.9	109.8
	A(22-25-26)	122.6	122.6
	A(29-28-37)	119.6	118.8
	A(28-29-30)	119.8	120.0
	A(28-29-31)	119.5	120.0
	A(28-37-35)	120.2	120.8
	A(28-37-38)	119.3	119.6
	A(30-29-31)	120.6	119.9
	A(29-31-32)	119.1	119.4
	A(29-31-33)	121.0	121.2
	A(32-31-33)	120.0	119.3
	A(31-33-34)	120.3	120.3
	A(31-33-35)	119.3	119.2
	A(34-33-35)	120.4	120.5
	A(33-35-36)	120.3	120.0
	A(33-35-37)	120.4	119.9
	A(36-35-37)	119.3	120.1
	A(35-37-38)	120.5	119.5
	A(41-40-42)	117.1	117.5
	A(40-42-43)	115.4	114.9
	A(40-42-75)	124.3	125.0
	A(43-42-75)	120.2	120.0
	A(42-43-44)	117.2	118.9
	A(42-43-45)	122.2	122.4

	A(42-75-61)	118.1	118.3
	A(44-43-45)	120.6	118.7
	A(43-45-46)	123.8	123.6
	A(43-45-59)	116.5	116.7
	A(46-45-59)	119.7	119.7
	A(45-46-47)	112.1	113.0
	A(45-46-51)	109.6	108.6
	A(45-46-55)	109.5	110.4
	A(45-59-60)	117.2	117.6
	A(45-59-61)	125.1	124.7
	A(47-46-51)	108.1	108.1
	A(47-46-55)	108.1	107.7
	A(46-47-48)	111.8	109.5
	A(46-47-49)	109.7	109.4
	A(46-47-50)	111.8	109.5
	A(51-46-55)	109.3	109.0
	A(46-51-52)	111.9	109.4
	A(46-51-53)	110.4	109.4
	A(46-51-54)	110.9	109.4
	A(46-55-56)	110.9	109.5
	A(46-55-57)	110.5	109.4
	A(46-55-58)	111.9	109.5
	A(48-47-49)	107.5	109.4
	A(48-47-50)	108.3	109.5
	A(49-47-50)	107.5	109.5
	A(52-51-53)	107.6	109.6
	A(52-51-54)	107.7	109.5
	A(53-51-54)	108.1	109.5
	A(56-55-57)	108.1	109.5
	A(56-55-58)	107.7	109.5
	A(57-55-58)	107.6	109.5
	A(60-59-61)	117.7	117.7
	A(59-61-62)	121.5	121.0
	A(59-61-75)	117.9	117.9
	A(62-61-75)	120.6	121.1
	A(61-62-63)	111.8	111.4
	A(61-62-67)	110.2	110.5
	A(61-62-71)	110.2	109.3
	A(63-62-67)	107.3	107.5
	A(63-62-71)	107.3	108.3
	A(62-63-64)	112.3	109.5
	A(62-63-65)	108.9	109.5
	A(62-63-66)	112.3	109.4
	A(67-62-71)	109.9	109.9
	A(62-67-68)	110.6	109.4
	A(62-67-69)	109.7	109.3

	A(62-67-70)	111.6	109.5
	A(62-71-72)	111.6	109.5
	A(62-71-73)	109.7	109.4
	A(62-71-74)	110.7	109.4
	A(64-63-65)	107.3	109.5
	A(64-63-66)	108.4	109.5
	A(65-63-66)	107.3	109.4
	A(68-67-69)	107.8	109.5
	A(68-67-70)	108.8	109.6
	A(69-67-70)	108.2	109.5
	A(67-70-4)	116.9	119.3
	A(72-71-73)	108.3	109.5
	A(72-71-74)	108.8	109.5
	A(71-72-4)	117.0	123.3
	A(73-71-74)	107.8	109.5

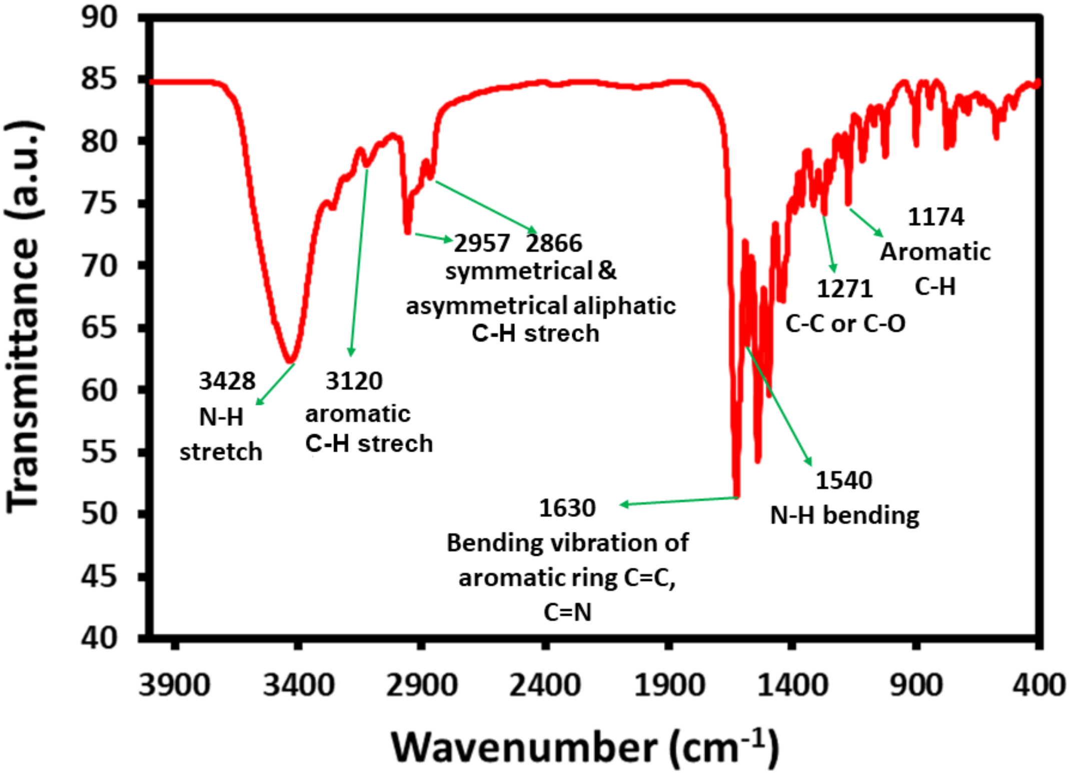

Figure S1. FTIR spectra of the studied Pt(II) complex.

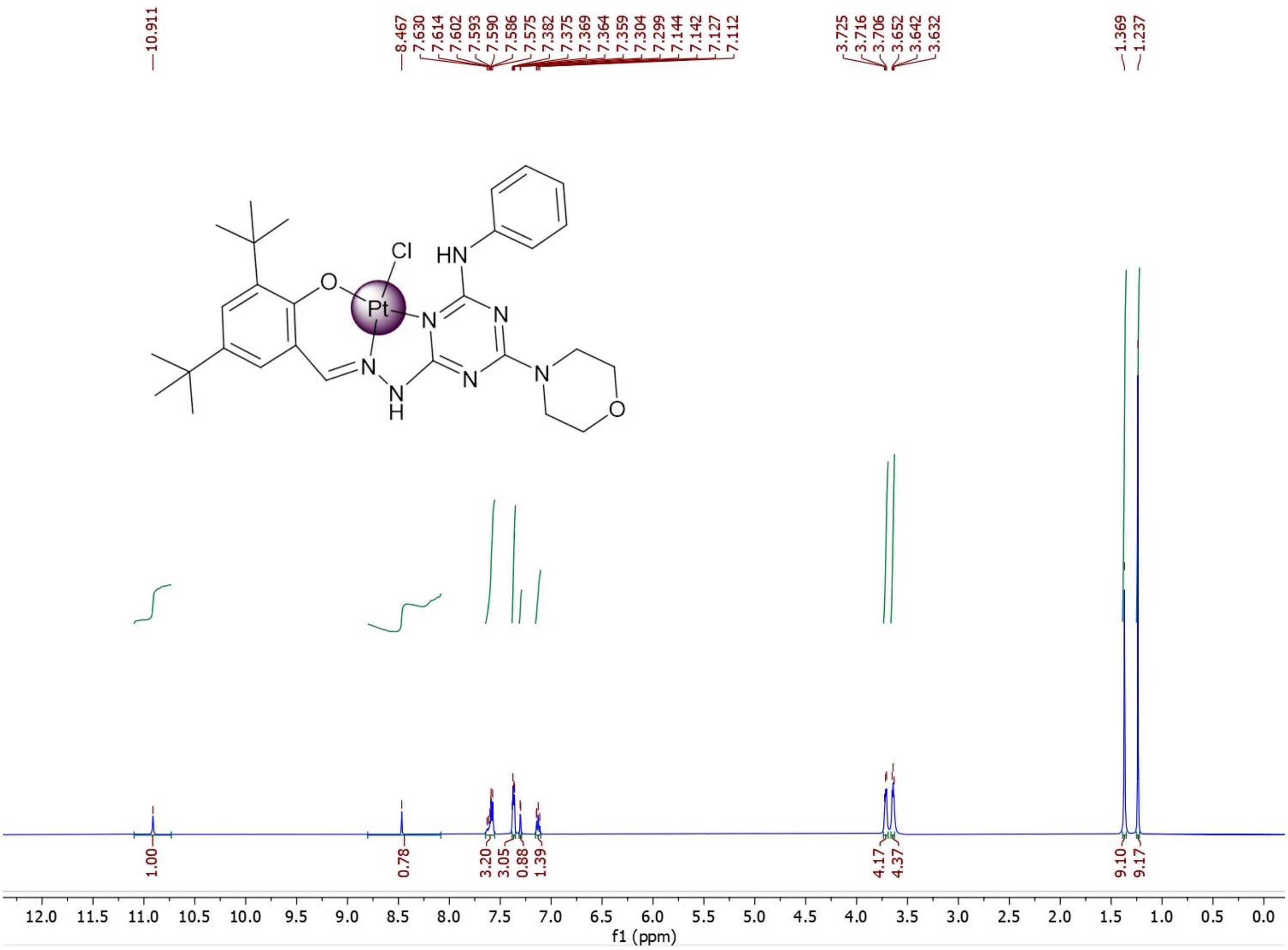

Figure S2. ^{1}HNMR spectra of the studied Pt(II) complex.

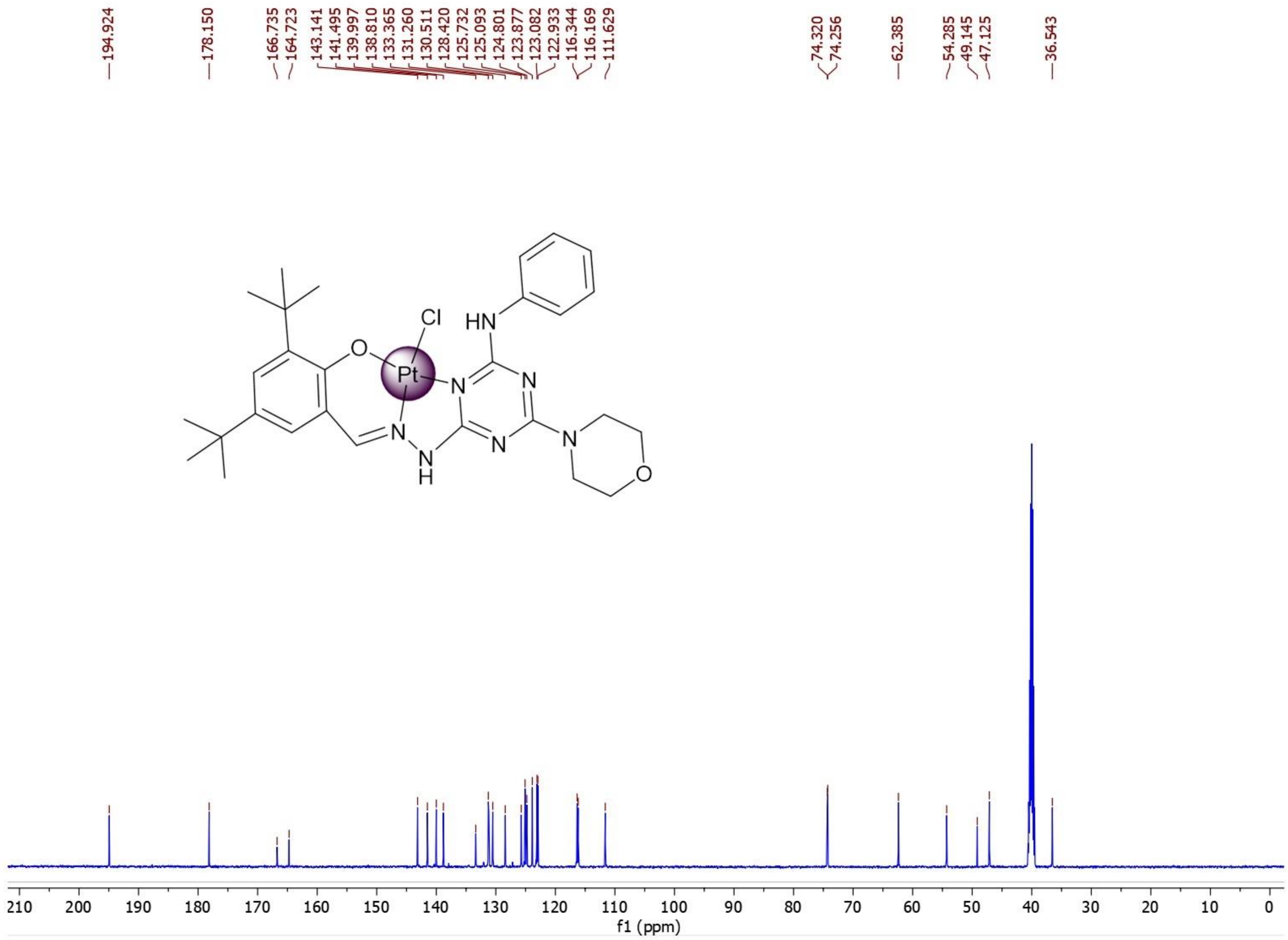

Figure S3. ^{13}CNMR spectra of the studied Pt(II) complex.
